# Modification of Peptide and Permeation Enhancer *In Vitro* Release Rates by Dispersion with a Gel-Forming Polymer

**DOI:** 10.1007/s11095-025-03870-y

**Published:** 2025-06-06

**Authors:** Pradnya Bapat, Sheena Lee Luy, Neha Panchabhai, Lynne S. Taylor

**Affiliations:** Department of Industrial and Molecular Pharmaceutics, College of Pharmacy, Purdue University, West Lafayette, IN 47907 USA

**Keywords:** copovidone, peptide, permeation enhancer, release rate

## Abstract

**Purpose:**

Herein, we evaluated the release properties of peptides when combined with a permeation enhancer (PE) as well as a gel-forming polymer.

**Methods:**

Octreotide was selected as a model hydrophilic peptide, while cyclosporine was chosen as a lipophilic peptide. The PEs studied were sodium decanoate (SD) and salcaprozate sodium (SNAC). To achieve synchronous release of the peptide and the PE, copovidone, a gel-forming polymer, was also included. Solid dispersions containing peptide, PE and polymer were prepared by dissolving all components in methanol followed by solvent removal. Dispersions were evaluated using powder X-ray diffraction. Surface normalized release rates of peptide, SNAC and copovidone alone and in combination were measured using Wood’s intrinsic dissolution rate apparatus.

**Results:**

Octreotide dissolved rapidly while amorphous cyclosporine release rate was essentially undetectable. The PEs and neat polymer also dissolved rapidly. However, the intrinsic dissolution rates of octreotide and SNAC differed by a factor of two. Addition of copovidone to the formulation led to synchronous release of octreotide and SNAC, controlling their release. Furthermore, both SNAC and SD enhanced the dissolution rate of the polymer, leading to very rapid release of the components from the ternary dispersion. Cyclosporine released well from dispersions when present at a very low concentration, with a deterioration in release performance being observed at higher drug loadings.

**Conclusions:**

Based on the findings of this study, inclusion of a gel-forming polymer may help synchronize the release of a hydrophilic peptide and a PE, which in turn may improve co-localization at the epithelial membrane.

**Graphical Abstract:**

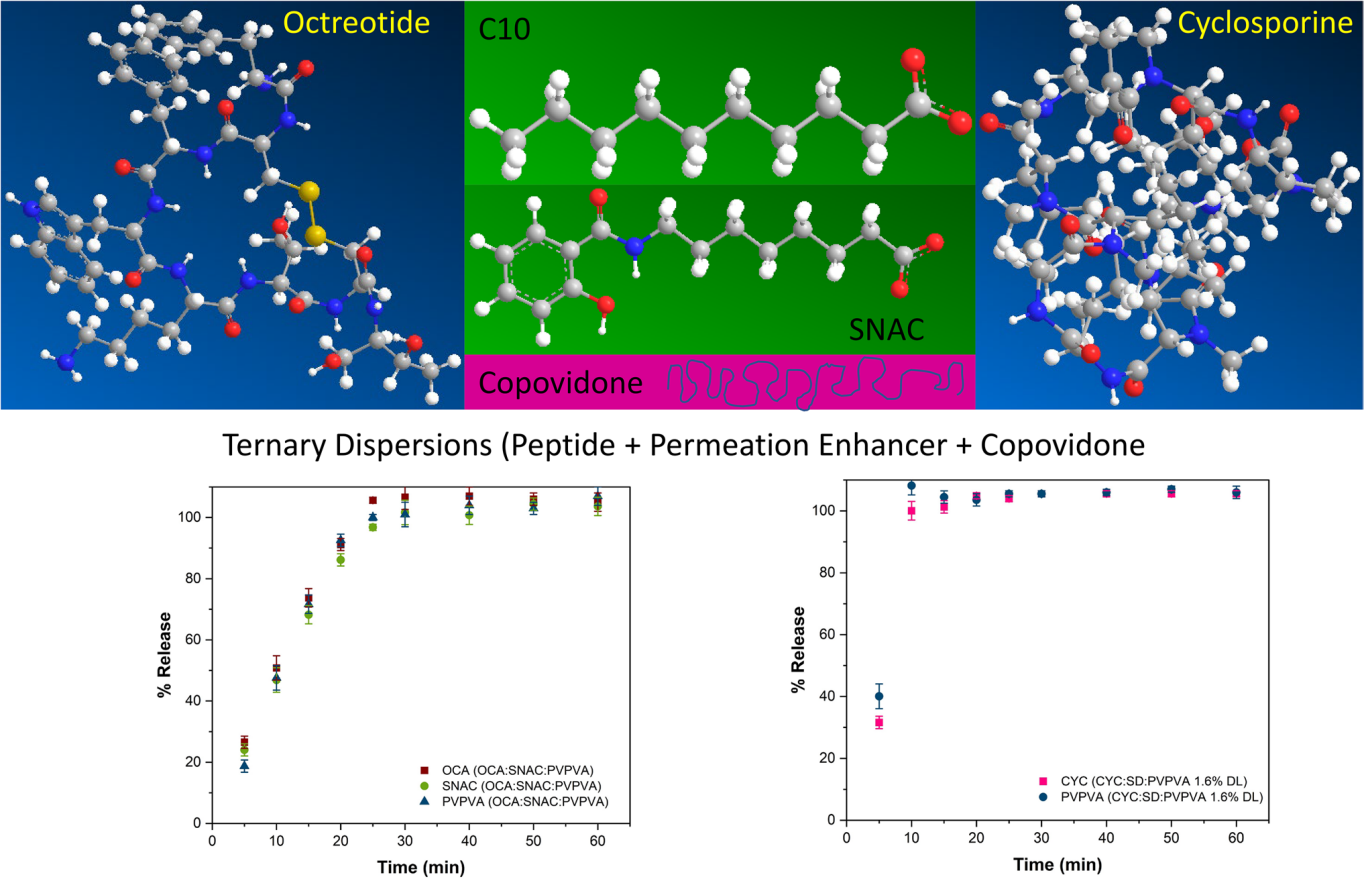

**Supplementary Information:**

The online version contains supplementary material available at 10.1007/s11095-025-03870-y.

## Introduction

With the recent approval of several products, notably Rybelsus® (semaglutide) in 2019, Mycapssa® (octreotide) in 2020, and Lupkynis® (voclosporin) in 2021, there is a resurgence of interest in formulation strategies for oral peptide delivery. This interest is also driven by the large number of peptides currently in clinical development [1]. All of the aforementioned recently approved peptide products are based on enabling formulation technologies, addressing sub-optimal physicochemical properties that lead to the poor oral absorption often observed for beyond-rule-of-five peptides. For semaglutide and octreotide, the peptides are poorly permeable due to their high molecular weight and hydrophilicity, and the formulations contain a permeation enhancer (PE). Semaglutide contains the PE, salcaprozate sodium (SNAC), as a tablet formulation, while octreotide is formulated as an enteric coated capsule with sodium caprylate (C8) as the PE, together with surfactants and lipids. Voclosporin is a modified form of cyclosporine and, like cyclosporine, is formulated as a self-emulsifying drug delivery system (SEDDS) by dissolving the drug in a mixture of ethanol, surfactants and lipids [2–4]. In the case of voclosporin, the SEDDS formulation is used to counteract the low aqueous solubility of the drug. [5] These enabling formulations achieve different extents of oral bioavailability. Even with PEs, semaglutide and octreotide achieve low oral bioavailability. The oral bioavailability of semaglutide when dosed with SNAC is 0.4–1% [6], while octreotide has a reported oral bioavailability of 0.7% [7]. The bioavailability of voclosporin is estimated as approximately 50% [5], consistent with its much higher permeability, and successful mitigation of its low solubility through the SEDDS formulation.

PEs have been studied for many years, and comprise a group of chemically diverse compounds, with different proposed mechanisms of action [7–17]. These mechanisms include membrane fluidization and disruption of tight junctions [10, 11, 14, 15, 18–33]. Ideal PEs effectively increase peptide permeability by rapidly and reversibly impacting gastrointestinal membrane properties. Reversibility is important to conserve the protective function of the gastrointestinal membrane. In general, it is known that high local concentrations of PEs are required to increase the membrane transport of hydrophilic peptides. This necessitates strategies that can deliver the PE to the absorption site, synchronously with the peptide. For the semaglutide formulation, it has been demonstrated that absorption occurs chiefly in the stomach, from an eroding formulation that exerts local effects on the gastric mucosa and fluids including buffering the pH to higher values [34]. In contrast, the octreotide formulation is enteric coated, to bypass the gastric environment and deliver the peptide to the small intestine.

Regardless of the site of absorption, co-localization of the peptide and PE, as well as high local PE concentrations are considered important for successful improvement of permeability [8, 11, 23, 28, 29, 35–42]. This has been shown in numerous studies, where comparisons have been made between simultaneous or staggered delivery of PE and peptides, whereby a short window of time exists for absorption of the peptide once the PE has exerted its effect on the membrane. [12, 13, 43, 44] Consequently, a challenge for oral drug delivery of peptides and PEs is the co-localization of peptide and PE at the membrane surface whereby the PE forms a sufficiently concentrated solution to perturb the membrane. This is challenging to achieve with a solid oral delivery system, especially when targeting the small intestine. In an intriguing study, Baluom *et al*. coformulated the poorly permeable small molecule, sulpiride, with the permeation enhancer, sodium decanoate (SD) and different grades of hydroxypropyl methyl cellulose (HPMC) [45]. The incorporation of HPMC was designed to synchronize the release of the drug and the PE over different time periods. *In vivo* studies following intra-intestinal administration demonstrated improved outcomes for synchronously releasing formulation relative to non-synchronously releasing systems. Furthermore, synchronous release over 1 h yielded improved absorption relative to slower synchronous release rates.

**Fig. 1:**
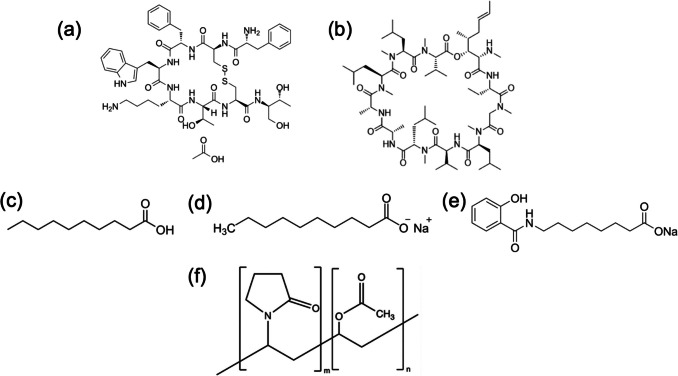
Chemical structures of (**a**) octreotide acetate (OCA), (**b**) cyclosporine (CYC), (**c**) decanoic acid (DA), (**d**) sodium decanoate (SD), (**e**) salcaprozate sodium (SNAC), (**f**) copovidone (PVPVA)

We and others have observed in numerous studies of amorphous solid dispersions of lipophilic small molecules that congruent (i.e. synchronous) release can be achieved with copovidone (PVPVA) formulations, whereby the polymer controls the release rate, and drug is released at a similar rate to the release rate of the neat polymer [46–51]. Given the anticipated need for advanced peptide formulations of both hydrophilic and hydrophobic peptides, combined with PEs, the goal of this study was to study the release properties of model peptides and PEs, alone and in combination with copovidone to determine if congruent release of peptide and PE can be achieved for these systems. Cyclosporine and octreotide were selected as model hydrophobic and hydrophilic peptides respectively, with structures shown in Fig. 1. SD and SNAC were identified as PEs of contemporary interest. Binary and ternary dispersions were prepared using solvent evaporation, and the resultant dispersions were characterized using powder X-ray diffraction. Surface normalized release rate measurements were performed using Wood’s dissolution apparatus.

## Materials

Octreotide acetate (OCA) was purchased from APIChem Technology (Zhejiang, China). Sodium decanoate (SD) and decanoic acid (DA) were supplied by Sigma Aldrich (MO, USA). Salcaprozate sodium (SNAC) was ordered from MedchemExpress (NJ, USA). Cyclosporine was purchased from Watson Laboratories, Inc (IL, USA). Poly vinylpyrrolidone-co-vinyl acetate (PVPVA) was supplied by BASF (Ludwigshafen, Germany) under the brand name Kollidon VA64. Ethanol (EtOH), methanol (MeOH), hydrochloric acid (HCl), trifluoroacetic acid (TFA), acetonitrile (ACN), sodium chloride (NaCl) sodium hydroxide (NaOH), sodium phosphate dibasic anhydrous (Na_2_HPO_4_), sodium phosphate monobasic monohydrate (NaH_2_PO_4_.H_2_O) were purchased from Fischer Chemicals (Fair Lawn, NJ, USA). Acid media of pH 1.6 was prepared by adding 2 g of NaCl to 0.9 L water and adjusting the pH with 1 N HCl. The composition of various phosphate buffer solutions is detailed in Table S1.

## Methods

### Analysis of Peptide, Permeation Enhancers and Polymer

For OCA and SNAC, an Ascentis^®^ Express (Sigma-Aldrich, St. Louis, MO) 90 Å C18 column with dimensions of 15 cm $\times$ 4.6 mm and particle size of 5 µm was used for high-performance liquid chromatography (HPLC) analysis. For CYC, a Zorbax Eclipse Plus (Agilent, Santa Clara, CA) C18 column with dimensions of 4.6 mm $\times$ 250 mm and a particle size of 5 µm was employed, while for copovidone, an A2500, Aqueous GPC/SEC column (300 × 8.0 mm) (P/N CLM3016, Malvern Panalytical, Worcestershire, UK) column was used. For OCA, 30:70 v/v 0.1% TFA in water and methanol was used as a mobile phase at 0.5 mL/min with an injection volume of 30 μL with ultraviolet (UV) detection at 210 nm. For CYC, 20:80 v/v water and acetonitrile was used as a mobile phase at 2.0 mL/min with injection volume of 100 μL with UV detection at 210 nm and the column was maintained at 75 °C. For SNAC, 20:80 v/v 0.1% TFA in water and methanol was used as a mobile phase at 0.5 mL/min with an injection volume of 20 μL and UV detection at 230 nm. For PVPVA, 80:20 v/v 50 mM pH 7.4 phosphate buffered saline (PBS) and methanol was used as a mobile phase at 0.5 mL/min with an injection volume of 50 μL and UV detection at 205 nm.

### Preparation of Solid Dispersions

Solid dispersions were prepared with SD: PVPVA, SNAC: PVPVA and DA: PVPVA at various w/w ratios. OCA:SD: PVPVA and OCA: SNAC: PVPVA at 1.6% drug loading (DL) were prepared by keeping the permeation enhancer to polymer ratio at 50:50 w/w. CYC: SD: PVPVA and CYC: SNAC: PVPVA ASDs were prepared at 1.6%, 5%, 10% and 20% DL with a 50:50 w/w ratio of permeation enhancer to polymer. For all dispersions, the components were added to a round bottom flask, dissolved in methanol and the solvent was evaporated using a rotary evaporator (Hei-VAP Core rotary evaporator, Heidolph Instruments, Schwabach, Germany) equipped with an Ecodyst EcoChyll S cooler (Ecodyst, Apex, NC, USA) with a water bath maintained at 45 °C. Dispersions were kept under vacuum overnight to remove excess solvent. They were then triturated using a mortar and pestle and stored in desiccator.

### Powder X-Ray Diffraction (PXRD)

PXRD was used to check the crystallinity of the dispersion powders. The diffractograms of neat OCA, CYC, SD, DA and SNAC as well their PVPVA-based dispersions were collected using a Rigaku SmartLab diffractometer (Rigaku corporation, Tokyo, Japan) with data collected at 0.5 deg/min over a 2θ range of 4–40°, a step size of 0.02° 2θ at 44 mA current and 40 kV voltage. Powder x-ray diffraction laboratory (PXDL) software was used to analyze the data.

### Surface Normalized Dissolution Rate Experiments

Surface normalized dissolution rate experiments were performed using an intrinsic dissolution rate (IDR) Wood’s apparatus (Agilent Technologies, Santa Clara, CA). Briefly 100 mg of dispersion was added to a die with a surface area of 0.5 cm^2^ and compressed using a Carver press (Carver, Wabash, IN) at 1500 psi with the pressure held for 1 min. The die was then attached to the rotating spindle at 100 rpm. Only one surface of the die was exposed to the buffer where experiments were performed in 100 mL of media in a jacketed beaker maintained at 37 °C. Samples were collected at regular intervals every 5 min for first 30 min and then every 10 min until 60 min. Samples were analyzed for various components using the HPLC methods described above. The surface area normalized release rate was calculated using Eq. 1.

1 $$R= \frac{k \times V}{S \times x}$$

where $k$ is the slope of the regression line, $V$ is the volume of dissolution medium (100 mL), $S$ is the surface area of the die exposed to the dissolution medium (0.5 cm^2^) and $x$ is the weight fraction of each component.

IDR experiments were performed on select dispersions in pH 1.6 media. A two-stage dissolution experiment was also performed by carrying out surface normalized dissolution in 100 mL of pH 1.6 media for one hour, followed by addition of 15 mL of concentrated pH 7.3 phosphate buffer to increase the pH to pH 6.8 and performing surface normalized dissolution for another hour.

### Particle Size Analysis Using Dynamic Light Scattering

The particle size of any colloidal species generated during surface normalized dissolution experiments at 37 °C was measured using a Zetasizer ULTRA by Malvern Instruments (Westborough, MA, USA) equipped with an Avalanche Photo Diode detector. Samples were taken at fixed time intervals during dissolution experiments with various dispersions in pH 1.6 acidic media and pH 6.8 phosphate buffer. The measurements were performed on filtered (0.2 μm nylon filter) and unfiltered samples in disposable polystyrene plastic cuvettes of 10 cm pathlength with samples maintained at 37 °C.

### Fluorescence Spectroscopy

Fluorescence spectra were obtained to probe potential interactions between various components after dissolution given that OCA and SNAC are both autofluorescent. Calibration curves of emission intensity as a function of concentration for OCA and SNAC were collected using a Shimadzu RF-5301pc spectrofluorometer (Kyoto, Japan) in the concentration range of 1–100 μg/mL. The excitation wavelength was 296 nm and emission in the range of 250 to 550 nm was detected. The excitation slit width was 10 nm and the emission slit width of 3 nm. Spectra of neat components at a fixed concentration were compared with the solution resulting from a dispersion dissolved in buffer to achieve the same concentration. Samples were also collected following release of various dispersions and spectra were obtained from these solutions.

### Self-Association of PEs in the Presence and Absence of PVPVA

To measure the self-association of SD, a stock solution of SD was prepared at 50 mg/mL and serial dilutions were prepared in the range of 0.1- 40 mg/mL in 50 mM pH 6.8 phosphate buffer. Pyrene was used as an environment-sensitive fluorescent probe which interacts with micelles/vesicles with a change in fluorescence spectrum. The final pyrene concentration added to each SD solution was 0.4 μg/mL and the ratio of fluorescence at I_1_ (372 nm)/I_3_ (383 nm) was plotted against the concentration of SD. All vials were equilibrated at 37 °C for 30 min prior to measurements taken with a Shimadzu RF-5301pc spectrofluorometer (Kyoto, Japan). The SD concentration where a decrease in the I_1_/I_3_ ratio was observed was taken as the critical vesicle concentration (CVC). The CVC in the presence of the polymer was evaluated in a similar way by adding 1 mg/mL of PVPVA to each sample and evaluating the pyrene fluorescence spectrum for SD concentration over the range of 0.1- 40 mg/mL, again in 50 mM pH 6.8 phosphate buffer.

Self-association of SNAC was assessed by measuring the intensity of the emitted fluorescence of SNAC at 414 nm at various concentrations over the range of 0–2 mg/mL in 50 mM pH 6.8 phosphate buffer using the fluorimeter described above. The CVC in the presence of polymer was measured by adding 1 mg/mL of PVPVA to each sample and measuring the SNAC intensity at 414 nm over the same range of concentrations in 50 mM pH 6.8 phosphate buffer.

### Dynamic Vapor Sorption

Dynamic water sorption experiments were performed using a DVS Resolution gravimetric sorption analyzer (Surface Measurement Systems, Allentown, PA). Water sorption profiles were collected by equilibrating all samples at 37 °C and 0% relative humidity (RH) for two hours. The RH was then raised from 0 to 95% RH in a stepwise manner with a step size of 5% RH. The change in mass over time was recorded and the system moved to the next step when the rate of change in mass was less than 0.002% min^−1^.

## Results

### Surface Area Normalized Dissolution Rate Experiments

**Fig. 2:**
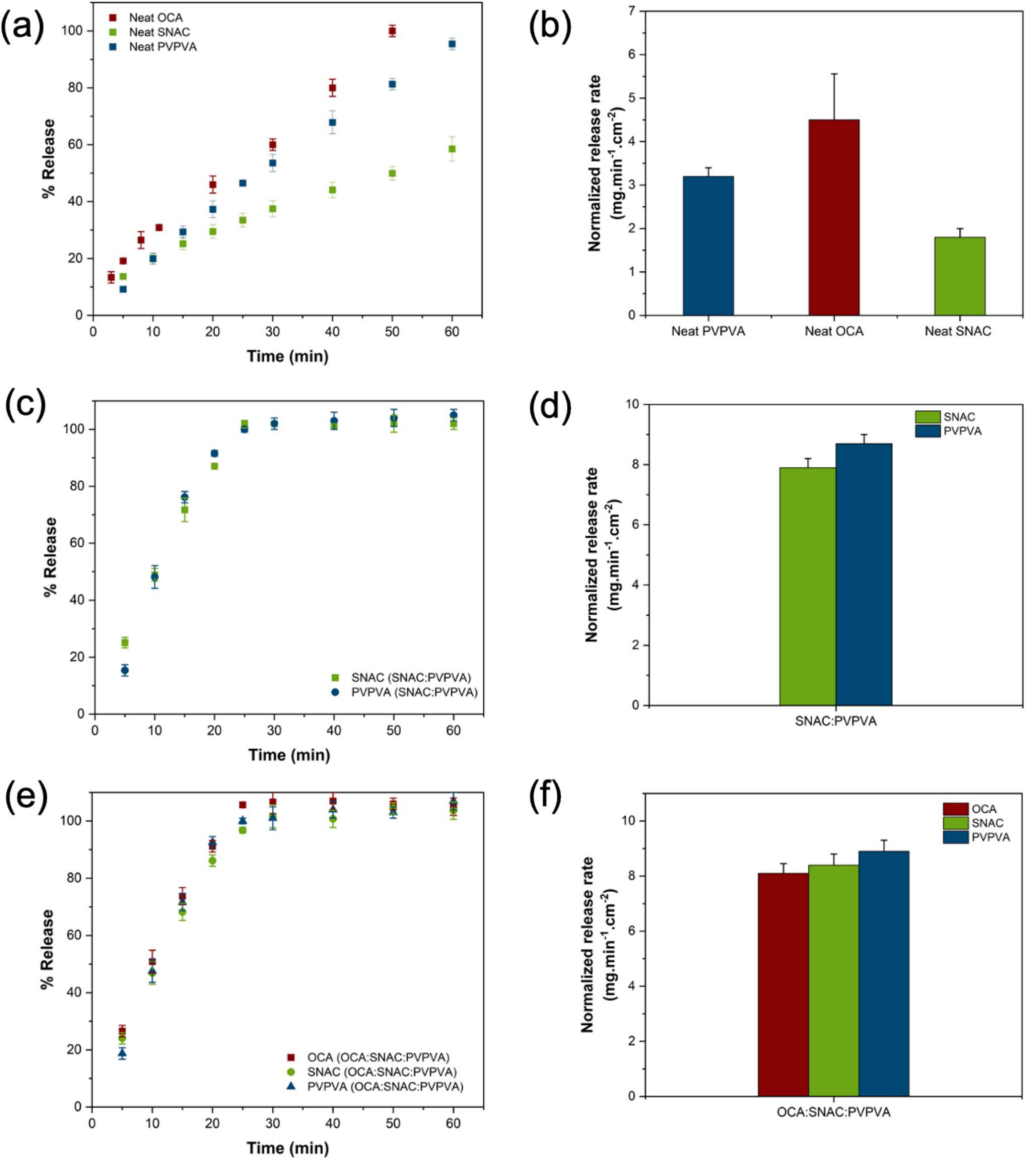
Percent release *vs.* time profiles for surface normalized dissolution and normalized release rates of (**a**, **b**) neat components, (**c**, **d**) SNAC, PVPVA from a SNAC: PVPVA 50:50 dispersion, (**e**, **f**) OCA: SNAC: PVPVA 1.6% DL dispersion in 50 mM pH 6.8 phosphate buffer. Error bars represent standard deviations, *n* = 3

**Fig. 3:**
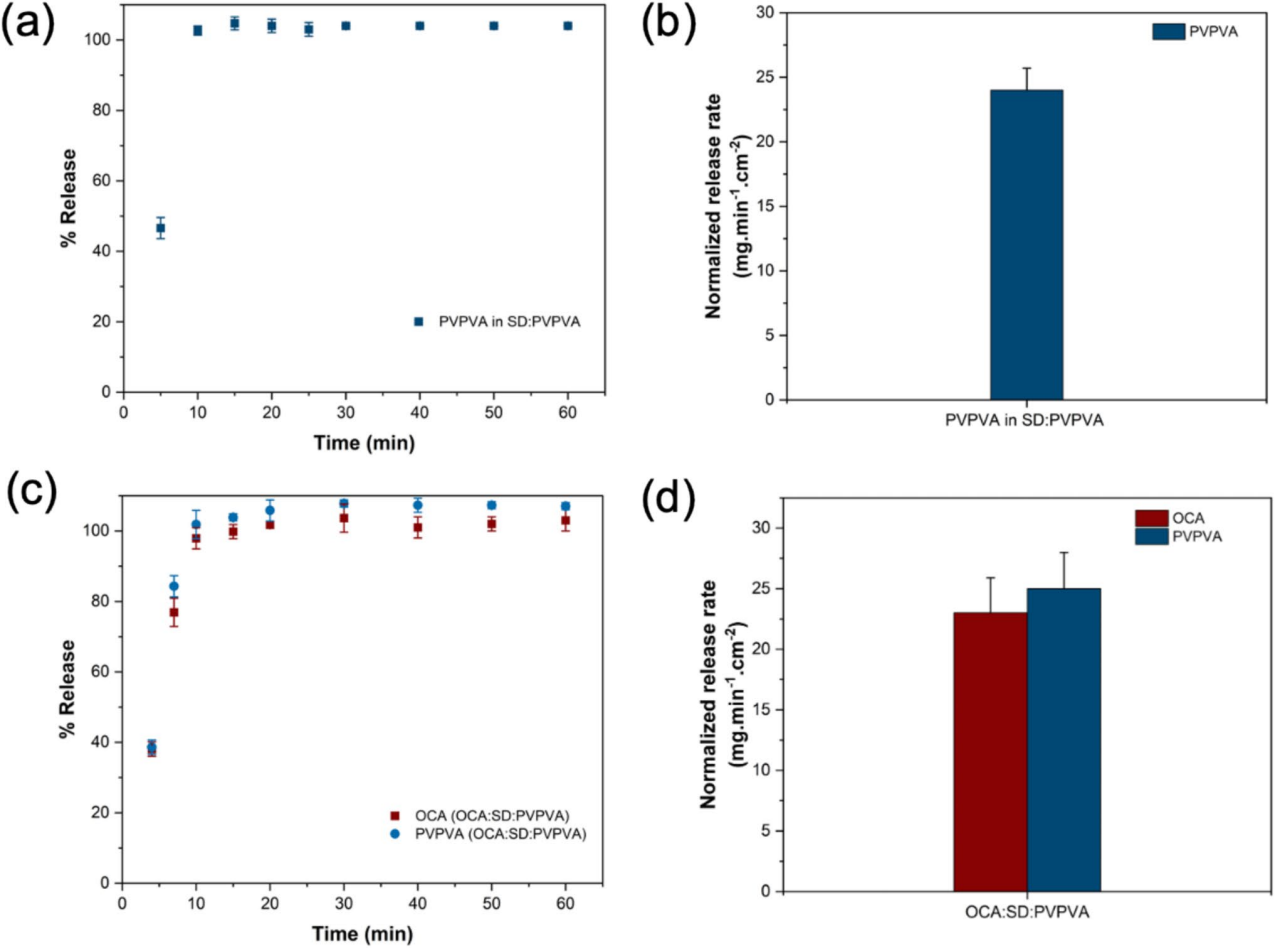
Percent release *vs.* time profiles for surface normalized dissolution and normalized release rates of dispersions of (**a**, **b**) PVPVA in SD:PVPVA 50:50, (**c**, **d**) OCA:SD:PVPVA 1.6% DL in 50 mM pH 6.8 phosphate buffer. Error bars represent standard deviations, *n* = 3

Figure 2 shows the percent release versus time profiles and surface area normalized dissolution rates in 50 mM pH 6.8 phosphate buffer of neat components, as well as various OCA formulations with SNAC, while Fig. 3 shows the corresponding data for formulations with SD. Surface normalized release rates were calculated using Eq. 1 applied to the linear portion of the curve. Of note, neat OCA has a rapid release rate (Fig. 2b) of 4.5 mg.min^−1^.cm^−2^, presumably driven by its high solubility and amorphous nature. Its intrinsic dissolution rate (IDR) is higher than that of neat PVPVA (~ 3 mg.min^−1^.cm^−2^). Neat SNAC also released quite fast (~ 2 mg.min^−1^.cm^−2^), but with a lower IDR than the peptide or polymer.

For SNAC dispersions, release of each of the components could be monitored. The surface area normalized release profiles and release rates of SNAC from a binary dispersion with PVPVA are shown in Fig. 2c and d, and it is apparent that the PE and polymer released at the same normalized release rates. The release rate of PVPVA was approximately doubled in the presence of SNAC, while the normalized release rate of SNAC increased by approximately fourfold (Fig. 2d). Thus, complete and simultaneous release was observed within 25–30 min for each component from 50:50 SNAC: PVPVA dispersions. The impact of different SNAC:PVPVA ratios was also evaluated (Figure S1). While the 10:90 SNAC: PVPVA dispersion showed a slightly slower release rate for each component (complete release within 40 min), dispersions of 20:80 SNAC: PVPVA or those with higher amounts of SNAC, showed rapid and congruent release of both components within 30 min. For a ternary dispersion of OCA: SNAC: PVPVA 1.6% DL, (Fig. 2e and f), it was observed that release of each component was more rapid than that of the neat components, and that each component released at the same relative rate. Thus, the release of the peptide and the PE could be controlled in the ternary system to be simultaneous. The normalized release rates of the components from the OCA dispersion with SNAC: PVPVA 50:50 were around 8 mg.min^−1^.cm^−2^.

The SD release rate could not be measured due to analytical constraints, but visual observation indicated that complete dissolution from the IDR die had occurred within 7–9 min for the neat SD solid, indicating that it had a faster intrinsic dissolution rate than SNAC. A binary dispersion of PVPVA and SD resulted in rapid polymer release (Fig. 3). For the neat polymer, approximately 60 min was required for complete release (Fig. 2a), while, somewhat surprisingly, this was reduced to around 10 min in the presence of SD (Fig. 3a). To further explore this effect, dispersions with different ratios of SD:PVPVA were prepared, and the polymer release rate was observed (Figure S2). It was found that polymer release was complete within approximately10 min when there was a minimum ratio of SD:PVPVA of 20:80. However, for the 10:90 SD: PVPVA dispersion, polymer release was complete by 25 min, approximately twice as fast as for neat polymer. These results suggest that the amount of SD in the formulation could be varied to control the polymer release rate.

Ternary dispersions of OCA:SD: PVPVA, containing 1.6% drug, also showed rapid release of OCA and PVPVA (and presumably SD as well, Fig. 3c and d). OCA and PVPVA released at the same normalized release rate. Thus, combining the SD with PVPVA led to a highly synergistic improvement in the release of each component. The normalized release rates of components from OCA dispersions with SD: PVPVA 50:50 were around 25 mg.min^−1^.cm^−2^. Thus, even the release of the highly hydrophilic OCA was enhanced relative to that of neat OCA when added to the SD-polymer blend.

**Fig. 4:**
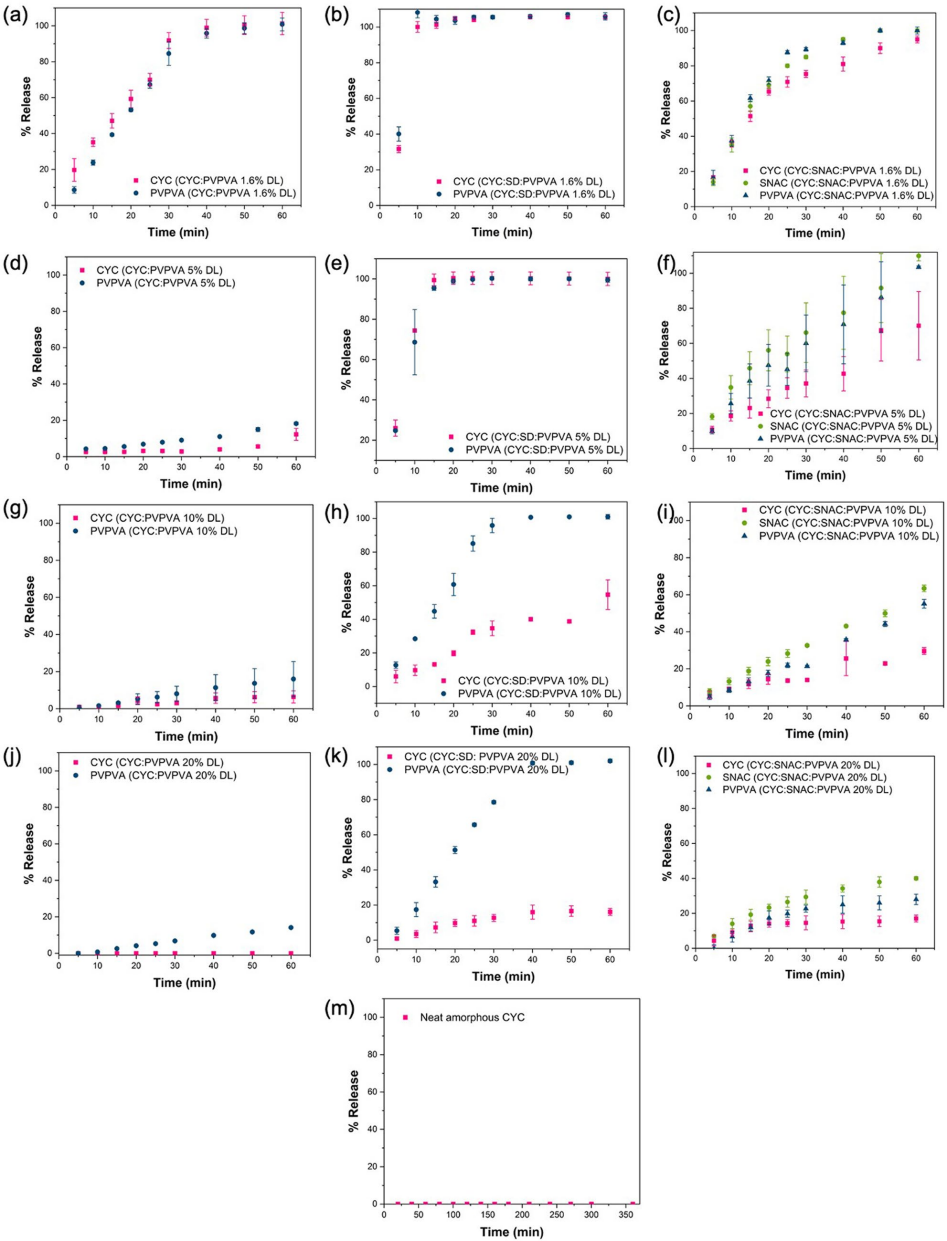
Percent release *vs.* time profiles for surface normalized dissolution of dispersions of (**a**) CYC: PVPVA 1.6% DL, (**b**) CYC:SD: PVPVA 1.6% DL, (**c**) CYC: SNAC: PVPVA 1.6% DL, (**d**) CYC: PVPVA 5% DL, (**e**) CYC: SD: PVPVA 5% DL, (**f**) CYC: SNAC: PVPVA 5% DL, (**g**) CYC: PVPVA 10% DL, (**h**) CYC: SD: PVPVA 10% DL, (**i**) CYC: SNAC: PVPVA 10% DL, (**j**) CYC: PVPVA 20% DL, (**k**) CYC: SD: PVPVA 20% DL, (**l**) CYC: SNAC: PVPVA 20% DL, and (**m**) neat amorphous CYC in 50 mM pH 6.8 phosphate buffer. Error bars represent standard deviations, *n* = 3

The release profiles for various dispersions of CYC with and without a PE are shown in Fig. 4. At a 1.6% DL, CYC released congruently with PVPVA from the binary dispersion, with release occurring over ~ 50 min (Fig. 4a). Addition of SD to the dispersion reduced the timeframe for complete release to ~ 10 min (Fig. 4b). For SNAC dispersions, release of each component occurred at the same normalized release rate, over a period of around 60 min, similar to the binary system (Fig. 4c). An increase in DL to 5% resulted in a large deterioration in release performance for the binary system (Fig. 4d) which was mitigated in the ternary dispersion with SD (Fig. 4e) and to some extent with SNAC (Fig. 4f). Release became progressively worse with an increase in DL from 10 to 20% in the binary systems (Fig. 4g and j). When SD was added to form a ternary dispersion, CYC release was improved relative to the corresponding DL binary systems (Fig. 4h,k), however, the release rate of PVPVA diverged from that of the drug. Furthermore, drug release was incomplete, reaching ~ 50% for the 10% DL dispersion and ~ 17% for the 20% DL dispersion. The higher DL ternary dispersions with SNAC showed different patterns of release (Fig. 4i and l). While CYC release was slower, both PVPVA and SNAC release were also retarded, with the relative release rates, while not identical, remaining more similar than in the case of SD dispersions. Finally, when considering neat amorphous CYC, the release rate was extremely slow, with less than 0.1% released after 6 h (Fig. 4m). For dispersions showing a large extent of incomplete release, PXRD patterns of the remaining dispersion after 60 min of dissolution testing were acquired. It was found that the remaining solid material was amorphous (Figure S3). Therefore, the plateau in CYC release was not due to crystallization of the peptide.

**Fig. 5:**
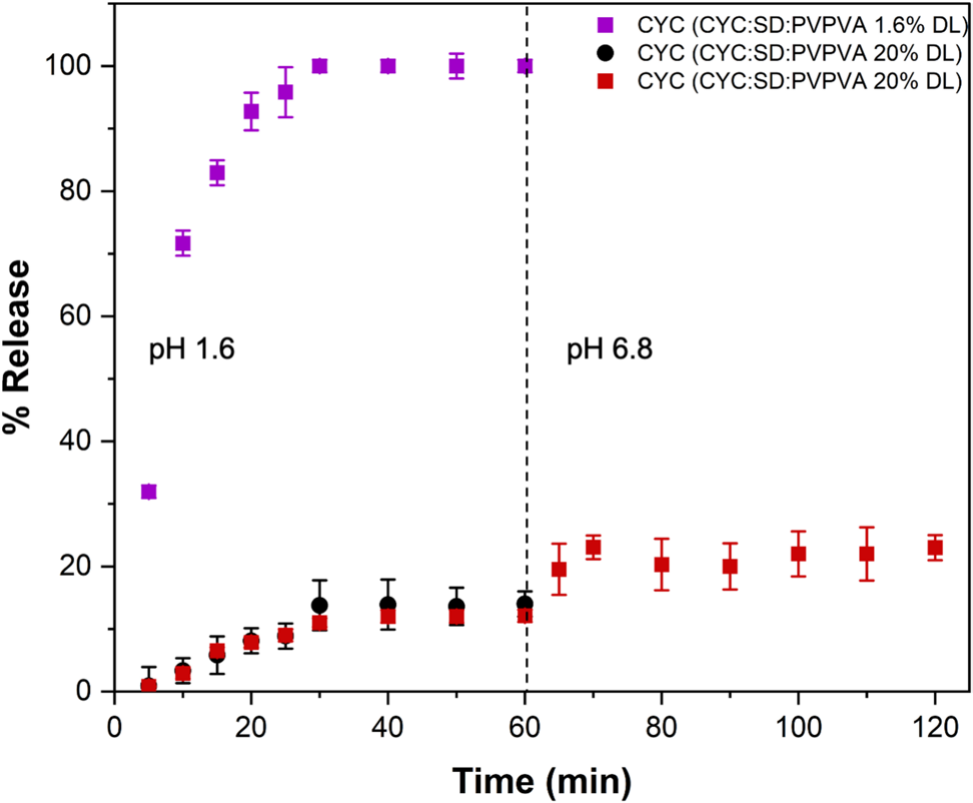
Percent release *vs.* time profiles for surface normalized dissolution of CYC:SD: PVPVA 1.6% and 20% DL in pH 1.6 and two-stage pH shift experiment from pH 1.6 to pH 6.8 for CYC:SD: PVPVA 20% DL. Error bars represent standard deviations, *n* = 3

Limited release experiments were also conducted in pH conditions that mimic the fasted-state gastric compartment. Figure 5 shows that complete release of drug from CYC:SD: PVPVA 1.6% DL dispersion occurred in pH 1.6 media, despite the low solubility of SD at this pH, while about 10% of the drug was released from the 20% DL ternary dispersion. SD is expected to convert to decanoic acid at pH 1.6 and may not fully dissolve, hence, a two-stage pH shift dissolution experiment was performed to see if additional CYC was released at higher pH. A small increase in the amount of CYC release was observed following media transfer to the higher pH condition. However, overall, for the 20% DL dispersion, the extent of CYC release was similar for the two stage experiment, as for the single stage experiments shown in Fig. 4.

### Powder X-Ray Diffraction (PXRD)

**Fig. 6:**
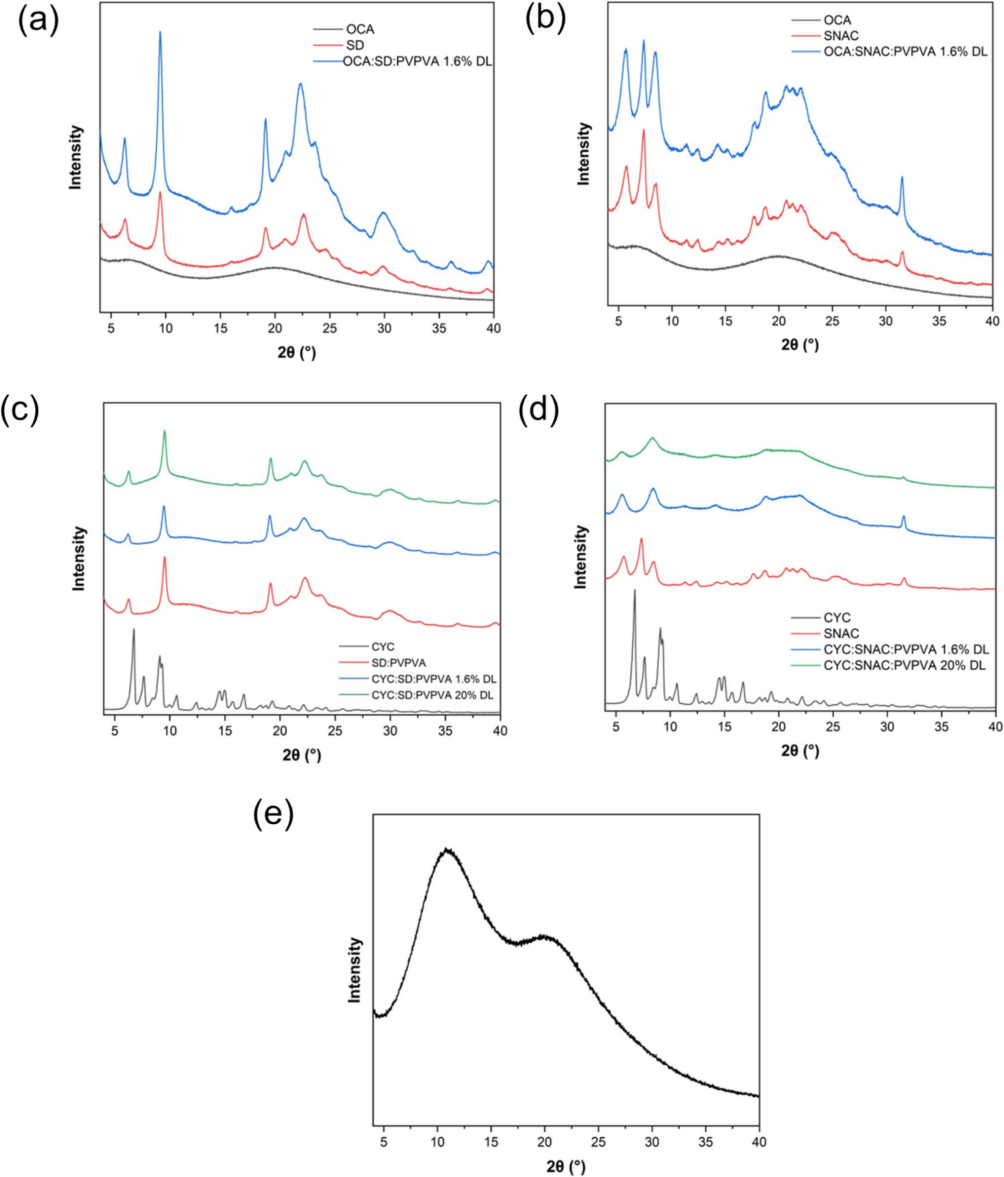
Powder x-ray diffractograms of (**a**) OCA:SD: PVPVA 1.6% DL, (**b**) OCA: SNAC: PVPVA 1.6% DL, (**c**) CYC:SD: PVPVA 1.6% and 20% DL, (**d**) CYC: SNAC: PVPVA 1.6% and 20% DL, (**e**) CYC: PVPVA 20% DL

PXRD studies were performed to evaluate the crystalline/amorphous nature of dispersions and neat components (Fig. 6). Neat OCA and PVPVA were amorphous whereas both PEs, as well as the as-received CYC were crystalline. The dispersion of CYC: PVPVA 20% DL without any PE was amorphous whereas all other dispersions had crystallinity arising from the PE, while OCA and CYC remained amorphous in the dispersions. SNAC crystallinity appeared reduced in the dispersions based on peak broadening while SD crystallinity appeared largely unchanged by the presence of the polymer.

### Size Analysis Using Dynamic Light Scattering

**Fig. 7:**
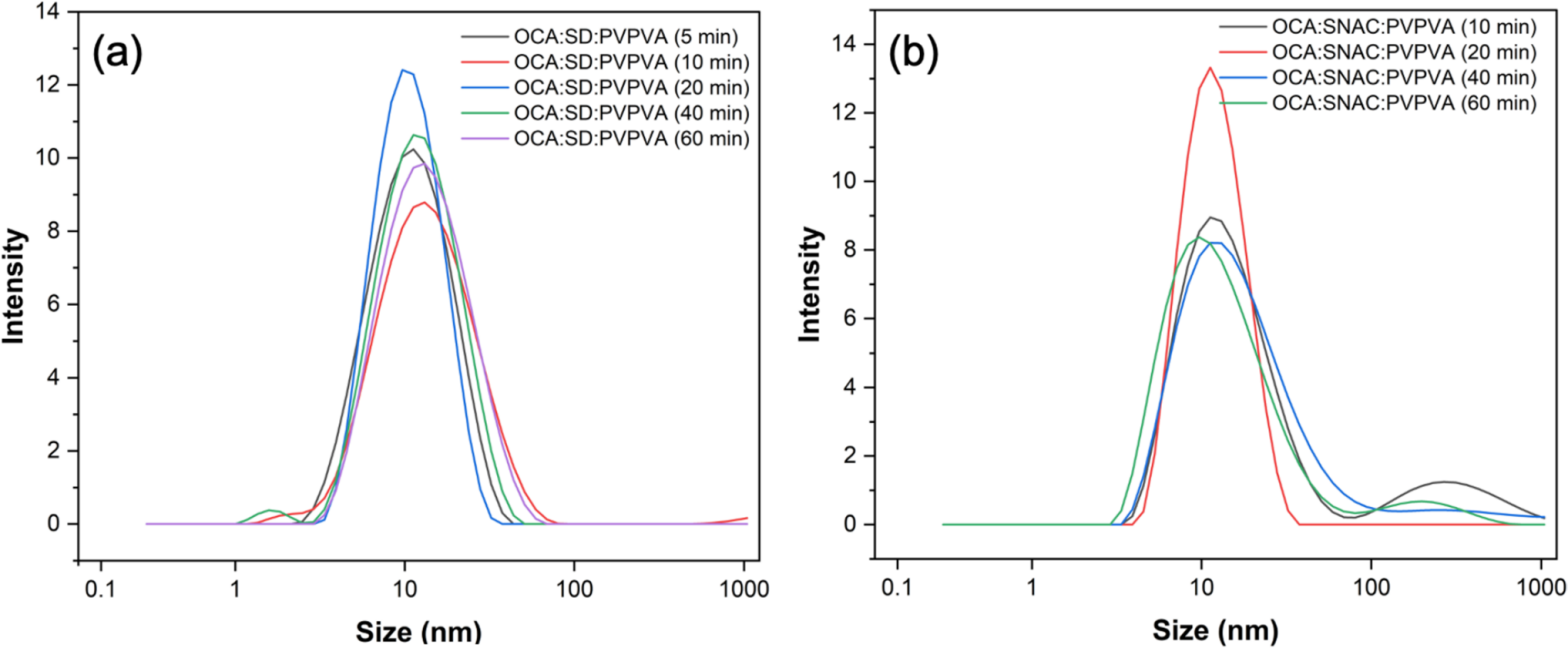
Particle size analysis using zetasizer at various time points during surface normalized dissolution of (**a**) OCA:SD: PVPVA 1.6% DL, (**b**) OCA: SNAC: PVPVA 1.6% DL

The sizes of colloidal species formed during surface normalized dissolution of OCA:SD: PVPVA 1.6% DL and OCA: SNAC: PVPVA 1.6% DL in pH 6.8 phosphate buffer at various time points for up to one hour are shown in Fig. 7. The average colloid size for the experimental duration was around 10 ± 5 nm for filtered samples. Unfiltered samples showed poor quality data with several peaks. For SD, this is consistent with previous reports of heterogeneous multilamellar vesicle formation under similar pH conditions. [52]

### Fluorescence Spectroscopy

**Fig. 8:**
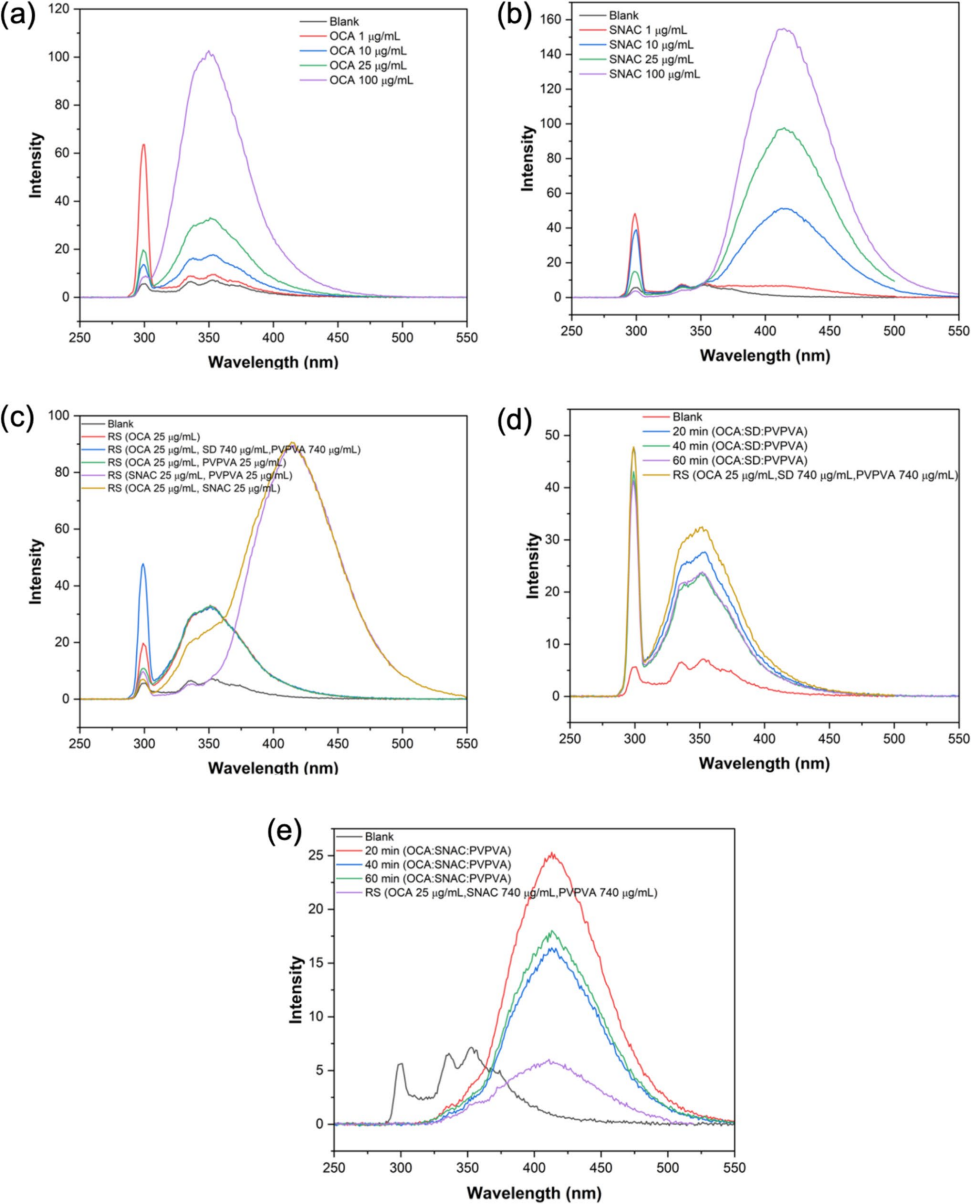
Fluorescence spectra of (**a**) various concentrations of OCA, (**b**) various concentrations of SNAC, (**c**) RS (reference solutions) of OCA, SNAC, PVPVA (**d**) samples from surface normalized dissolution of OCA:SD:PVPVA in pH 6.8 phosphate buffer with expected concentrations of 16 μg/mL OCA, 490 μg/mL SD, 490 μg/mL PVPVA achieved after 10 min release compared with RS of OCA, SD and PVPVA, (**e**) samples from surface normalized dissolution of OCA:SNAC:PVPVA in pH 6.8 phosphate buffer with expected concentrations of 16 μg/mL OCA, 490 μg/mL SNAC, 490 μg/mL PVPVA achieved by 25 min dissolution compared with RS of OCA, SD and PVPVA

Both OCA and SNAC are autofluorescent compounds (Fig. 8). The fluorescence spectra of OCA and SNAC at various concentrations are shown in Fig. 8a and b respectively. An increase in fluorescence intensity with concentration over the concentration range studied (0–100µg/mL) was observed for both compounds. The emission maximum for OCA was observed at around 350 nm, while for SNAC, the peak was at 414 nm. The fluorescence spectrum of OCA from dissolved OCA:SD: PVPVA or OCA: PVPVA dispersions overlaps the neat OCA spectra at an equivalent concentration. This likely suggests an absence of interactions between OCA with either SD or PVPVA at the concentrations tested. For OCA: SNAC: PVPVA dissolved in pH 6.8 buffer, the OCA fluorescence intensity was notably quenched relative to that of OCA alone. Similar observations were found for samples taken from surface normalized dissolution experiments at various time points, where the OCA fluorescence was highly quenched by the presence of higher SNAC concentrations. Moreover, at higher concentrations, SNAC fluorescence was also reduced relative to at lower concentrations, as can be seen from a comparison of the spectra in Fig. 8b and e. The concentration impact on SNAC fluorescence intensity was investigated in more detail.

### Self-association of PEs

**Fig. 9:**
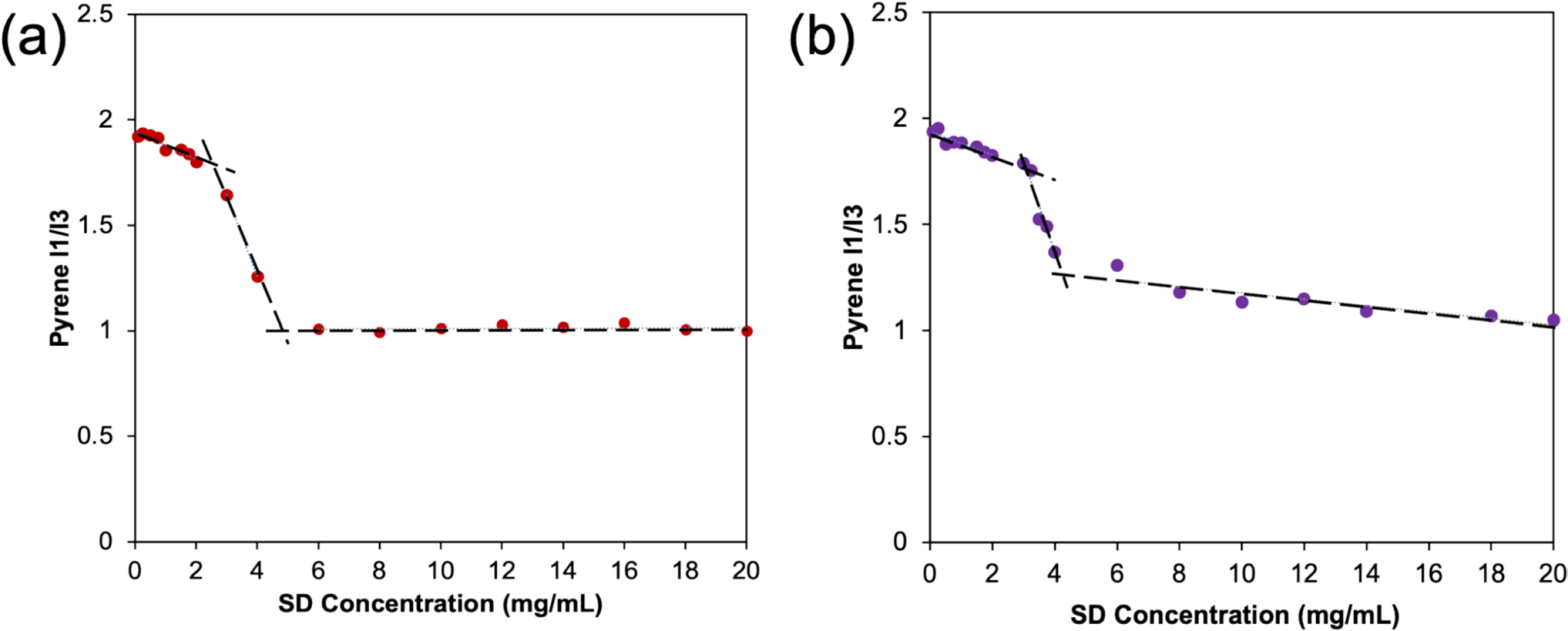
(**a**) CVC of SD, (**b**) CVC of SD in presence of 1 mg/mL PVPVA in 50 mM pH 6.8 phosphate buffer

Figure 9a shows the pyrene I_1_/I_3_ ratio as a function of SD concentration. A decrease in the ratio was observed at around 2.7 mg/mL, corresponding to the critical vesicle concentration (CVC) of SD in 50 mM pH 6.8 phosphate buffer. Figure 9b shows the corresponding data in the presence of 1 mg/mL of PVPVA, where a decrease in pyrene I_1_/I_3_ was observed at around 2.9 mg/mL. Thus, the presence of PVPVA does not appear to notably change the critical vesicle concentration of SD under these conditions. The value for the SD CVC is in good agreement with previously reported values at similar pH conditions. [52]

**Fig. 10:**
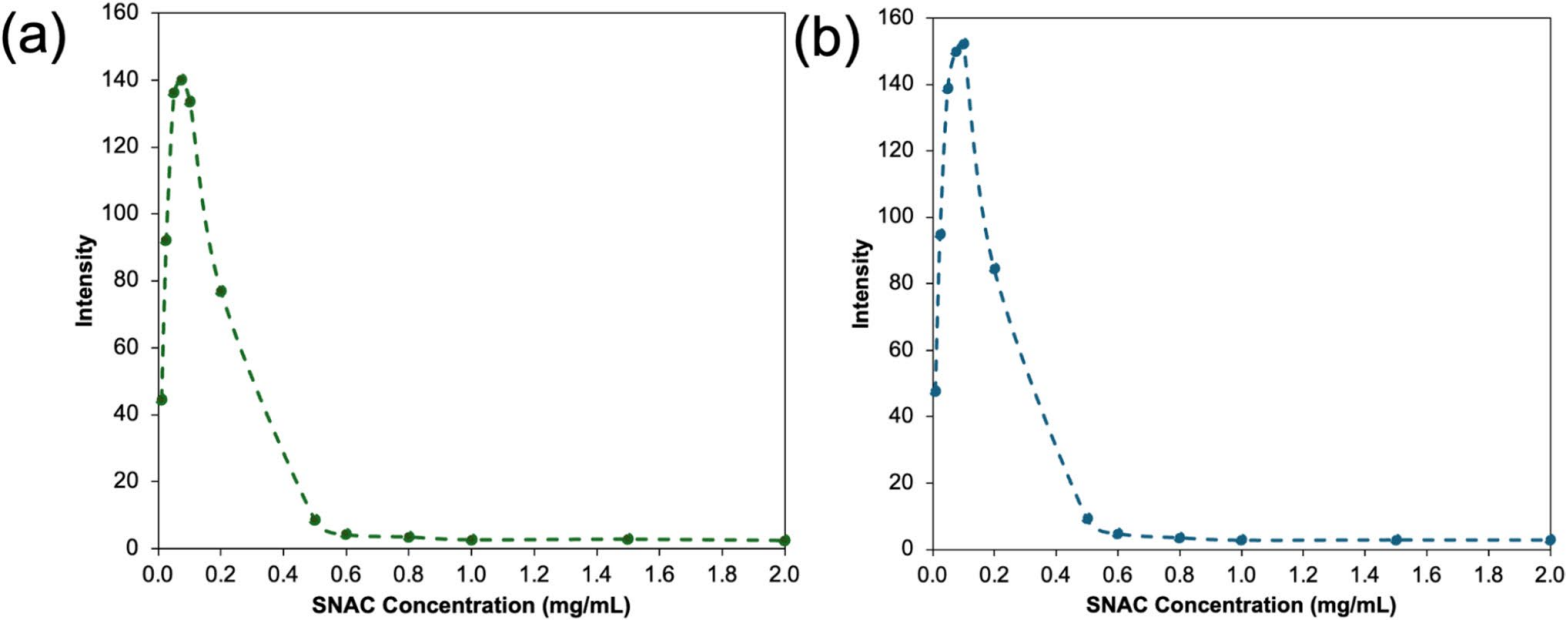
Fluorescence intensity as a function of SNAC concentration in the (**a**) absence and (**b**) presence of 1 mg/mL PVPVA, 50 mM pH 6.8 phosphate buffer

Figure 10 shows the fluorescence intensity of SNAC as a function of concentration in the presence or absence of PVPVA. The fluorescence intensity increases with concentration in the low concentration range, reaches a maximum and then decreases with increasing concentration, suggesting quenching due to self-association. The concentration where the maximum in the fluorescence intensity was observed was taken as the concentration where aggregation commenced. This concentration was not impacted by the presence of the polymer and was around 0.1 mg/mL.

### Dynamic Vapor Sorption

**Fig. 11:**
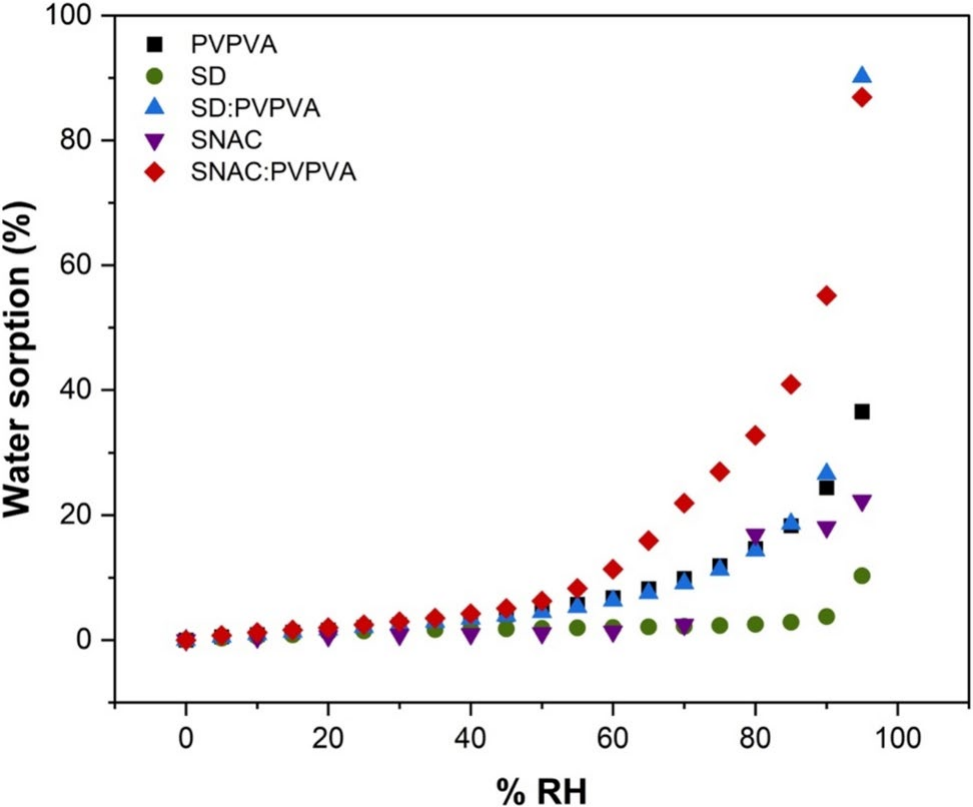
Water sorption profiles of neat PVPVA, SD, SD: PVPVA 50:50 w/w, SNAC, SNAC:PVPVA 50:50 w/w at 37 °C from 0 to 95% RH in a stepwise manner

Figure 11 shows water vapor sorption profiles for various samples at 37 °C. SD: PVPVA sorbed the most water, about 90% at 95% RH, while the SNAC: PVPVA dispersion acquired about 86% water under the same conditions. Neat SD sorbed approximately 10% water, SNAC gained about 22% whereas PVPVA absorbed about 37% water. The water sorption of the binary permeation enhancer: polymer dispersions therefore exceeded the amount of water predicted based on adding the sorption properties of the individual components.

## Discussion

Effective implementation of PEs requires co-localization of the PE and drug at the absorption site [12, 35, 53–60]. Furthermore, extensive studies have shown that the effectiveness of a PE is highly dependent on its concentration [8, 43, 61–64]. Studies demonstrating the link between PE concentration and absorption enhancement have been performed under highly controlled conditions. These typically involve rodent intestinal perfusion studies using solutions of known PE concentration, [17, 37, 57, 60, 65–67] or intra-intestinal administration of the formulation at a specific location. [16, 44, 68–70] During oral dosing, the fate of the dosage form is much less controlled, and hence achieving the optimum concentrations of PE and drug at the absorption site is much more challenging. Some of these challenges include the harsh gastric environment which may destroy the peptide, or lead to peptide but not PE solubilization, as well as dilution by secretions and variations in gastrointestinal motility which impact mixing extent and distribution of components. If the peptide and PE have different solubilities, then they will dissolve at different rates, potentially impacting their ability to co-localize at the absorption site. Importantly, PEs such as SD are rapidly absorbed (within 15–30 min) [1, 10, 16, 29, 69, 71–75], creating additional challenges for achieving favorable conditions for enhanced drug absorption and further emphasizing the need for synchronous drug and PE delivery at a rate sufficient to achieve an effective local PE concentration. For SD, various studies have suggested that a concentration of at least 10 mM or higher at the membrane surface is required. [76]

Much effort has been directed to exploring new PEs as well as elucidating the mechanism of action of established PEs. However, the release properties of components from solid oral dosage forms containing PEs is also an important topic known to have impact. In a seminal study, Rubenstein and coworkers prepared eroding matrix as well as immediate release tablets containing sulpiride, a poorly absorbed drug, and SD [77]. They demonstrated that enhanced drug absorption was afforded following rat intrajejunal administration using eroding tablets where drug and PE had synchronized release, relative to tablets where the PE released faster than the drug. Additionally, more rapid synchronized release yielded higher absorption extents than slower synchronized release. Their study thus demonstrated the importance of simultaneous release of drug and PE, as well as the timeframe of the release process. Likewise, it is generally considered important to achieve concurrent release of peptide and PE to achieve local high concentrations [12], although the optimized release rate is not clear, and is likely to depend on both the site of delivery, as well as the peptide and PE utilized in the formulation. Tran *et al*. studied the *in vitro* dissolution and *in vivo* oral absorption of a dual agonist peptide comprised of a glucose-dependent insulinotropic polypeptide (GIP) and glucagon-like peptide-1 (GLP-1) using eroding formulations containing either C10 or SNAC designed to target the gastric compartment [78]. Various formulations were prepared with “slow” and “fast” release rates. For SNAC, fast releasing tablets showed a trend towards improved peptide absorption, while for C10, a fast releasing capsule provided poorer absorption than a more slowly releasing tablet. These results may point towards the benefits of achieving a high localized concentration by dosing an eroding tablet formulation which releases peptide and PE over a specific timeframe, where that timeframe is not yet elucidated. When the intestinal compartment is targeted for peptide absorption, enteric coated dosage forms are often used [79]. Tyagi *et al*. studied various enteric coated particulate formulations containing a GLP-1 agonist and PE, where some of the formulations contained a mucoadhesive polymer [21]. Addition of a mucoadhesive polymer was not found to improve absorption. Moreover, delivery to the distal small intestine through selection of the appropriate enteric coating polymer was found to yield the best absorption, highlighting the importance of targeting the optimum absorption location. In a follow up study, the same group evaluated a variety of tablet formulations, targeted to release the peptide and PE at the optimum absorption site comparing immediate release (IR) and controlled release (CR) formulations, although no dissolution studies were reported [80]. While the peptide bioavailability and absorption variability was altered by formulation factors, no clear differentiation between immediate and controlled release formulations was discernable; IR and CR formulations with comparable *in vivo* performance were identified. Although these studies highlight the importance of peptide and PE release rates to *in vivo* absorption, it is clear that target release profiles remain to be elucidated. The utility of fundamental investigations of release processes, and the use of excipients to modify release rates has long been recognized in the delivery of small molecule therapeutics, motivating the approaches evaluated herein.

When considering conventional immediate release formulations, the relative dissolution rates of the peptide and PE will depend on their solubility in surrounding media (both in the unstirred water layer adjacent to the dissolving particle, as well as in the bulk medium), their relative surface areas, and their diffusion coefficients. From the data shown in Fig. 2b, it is apparent that OCA dissolves approximately two times as fast as SNAC under the experimental conditions employed. OCA is a highly hydrophilic peptide, with a rapid dissolution rate. In contrast, CYC is a much more hydrophobic peptide, and the IDR data shown in Fig. 4m illustrates a very slow release rate. Therefore, the relative dissolution rates of peptides versus PEs from simple formulations will be highly dependent on their individual characteristics and are unlikely to be similar. For the OCA: SNAC system, for comparable particle sizes, it would be anticipated that the peptide would dissolve faster than the PE when combined in an immediate release tablet, designed to release under intestinal conditions whereas the converse would be true for CYC: SNAC. In both instances, this difference in release rates is likely to impact the effectiveness of the PE at improving peptide membrane permeability. In particular, slower release of the peptide relative to the PE is likely to be an issue. Remarkably, forming a dispersion with PVPVA eliminates the anticipated differences in release rates, whereby each of the three components releases at the same, rapid normalized rate. Furthermore, when the peptide is present at a very low weight percent, as would be typical for a highly potent peptide, little difference is seen in release rate for OCA versus CYC, eliminating the impact of peptide solubility. When the percentage of the lipophilic peptide increases, the release rate and extent from the SNAC: PVPVA dispersion is, however, reduced although release is still rapid, albeit incomplete. Similar, rapid release rates were observed for dispersions containing the two peptides when formulated with SD and PVPVA, suggesting that the use of PVPVA to achieve synchronous release of peptide and PE can be applied to different types of PEs. Interestingly, PVPVA was able to synchronize release of peptide and PE, even though the PE was in crystalline form in the dispersion. This can likely be attributed to the gel-forming properties of PVPVA. Extensive recent studies have shown that this polymer forms a gel layer of > 200 µm thickness when immersed in water [81–83]. If the PE and peptide are trapped in the gel layer, then gel layer erosion will control the release of all components. This is likely the main mechanism behind the observed synchronous release of components at low peptide loadings. Further, coformulation of the polymer and PE led to synergistic increases in release rate for all components, that could be tailored to some extent by varying the PE:polymer ratio. Given that there are other gel-forming polymers that may also synchronize release, for example polyvinylpyrrolidone which comes in a variety of molecular weight grades with differing erosion rates [84], this approach lends itself to achieving different release rate time frames for peptides and PEs. The mechanism of the enhanced release rate of the PEs in the presence of PVPVA (and vice versa, Fig. 4) requires further investigation, but is likely due to enhanced water sorption in the binary mixture (Fig. 11). This would increase the rate of gel erosion, and hence the rate of release of all of the components. This enhancement in release rate was confirmed for PVPVA and SNAC dispersions (Fig. 2b and d). Maher and Brayden discussed the importance of co-presentation of the peptide and PE at the membrane surface [12]. One scenario is to use a liquid filled capsule, such as the commercial OCA formulation. For solid oral dosage forms, they postulate that the same bolus effect as that achieved by a liquid formulation could be afforded by a solid dosage form that dissolved within 5–15 min. Remarkably, this is readily achieved with the SD: PVPVA dispersion, where complete dissolution from the IDR apparatus is achieved within 10 min, despite the limited surface area exposed to the release medium. The dispersion approach utilized herein thus provides the advantages of a solid dosage form, coupled an extremely rapid and synchronous solubilization process.

## Conclusions

The observations of this study are extremely promising in terms of modulating and synchronizing the release of PE and peptides from oral formulations. While we have employed PVPVA as an example of a gel-forming polymer with a relatively rapid release rate in neat polymer form, there are many other pharmaceutically acceptable gel-forming polymers that could also be used for this strategy. These polymers could afford faster or slower release, depending on the desired *in vivo* performance and the properties of the polymer employed. This opens up many potential formulation avenues to achieve optimized release of peptides and PEs, with the ultimate goal of achieving improved peptide absorption.

## Supplementary Information

Below is the link to the electronic supplementary material.

Supplementary file1 (DOCX 4869 KB)
