# Supplementary material for: Modification of Peptide and Permeation Enhancer In Vitro Release Rates by Dispersion with a Gel-Forming Polymer
Source: Pharm Res. 2025 Jun 6;42(6):1003–20. doi: 10.1007/s11095-025-03870-y (PMC12222406; doi:10.1007/s11095-025-03870-y)
Supplement: Supplementary file 1 — Supplementary file1 (DOCX 4869 KB) [file 11095_2025_3870_MOESM1_ESM.docx]

Supporting information for the manuscript entitled

**Modification of Peptide and Permeation Enhancer Release Rates by Dispersion with a Gel-Forming Polymer**

***Pradnya Bapat,^1^ Sheena Lee Luy,^1^ Neha Panchabhai,^1^ Lynne S. Taylor.^1^****

1. Department of Industrial and Molecular Pharmaceutics, College of Pharmacy, Purdue University, West Lafayette, Indiana 47907, United States

*Correspondence: Lynne S. Taylor
 Telephone: +1-765-496-6614; Fax: +1-765-494-6545)

Email address: [lstaylor@purdue.edu](mailto:lstaylor@purdue.edu)

**Table S1. Composition of various phosphate buffer solutions**

| buffer type | NaOH  (g) | NaCl  (g) | Na_2_HPO_4_  (g) | NaH_2_PO_4_.H_2_O  (g) | water q.s.  (mL) |
| --- | --- | --- | --- | --- | --- |
| 0.57 M pH 7.3 concentrated phosphate buffer | 17.4 | 11.1 | 0 | 79 | 1000 |
| 50 mM pH 6.8 phosphate buffer | 0 | 0 | 3.468 | 3.529 | 1000 |


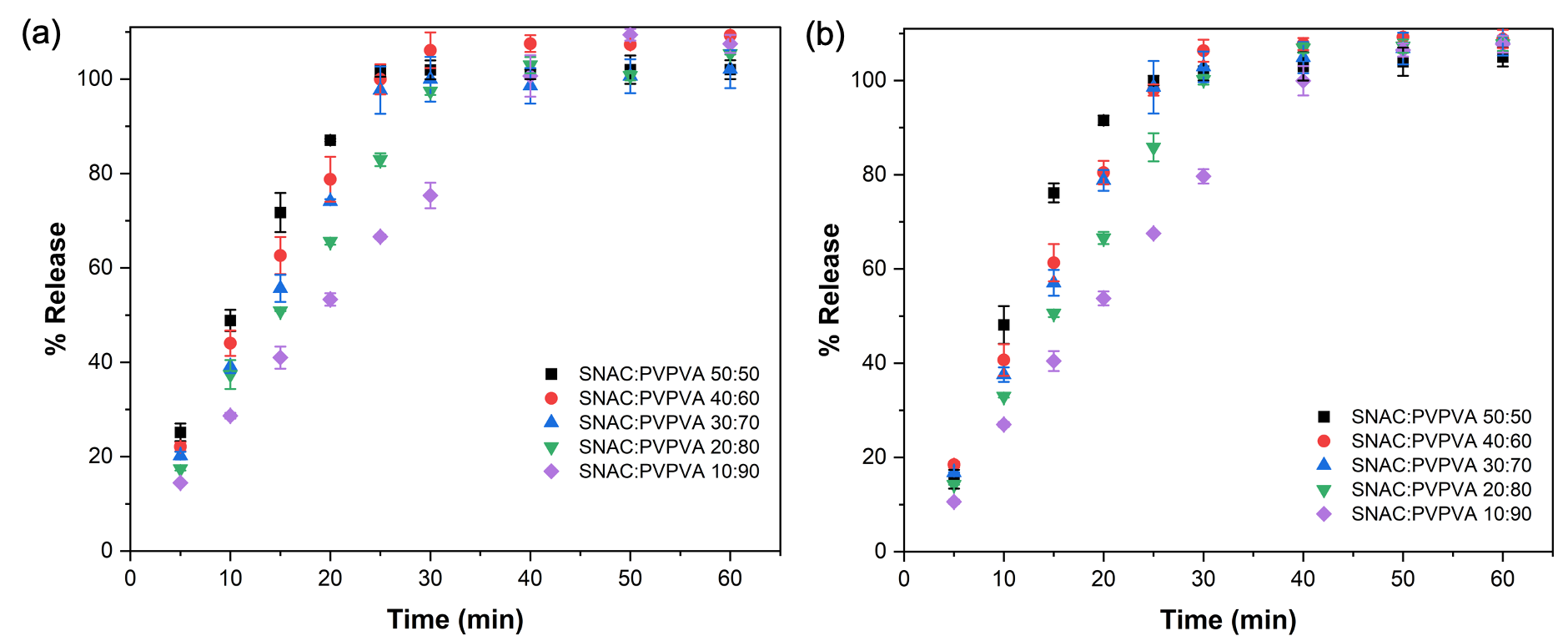


**Figure S1. Percent release vs. time profiles for surface normalized dissolution for (a) SNAC, (b) PVPVA from various w/w ratios of SNAC:PVPVA dispersions. Error bars represent standard deviation, n = 3.**


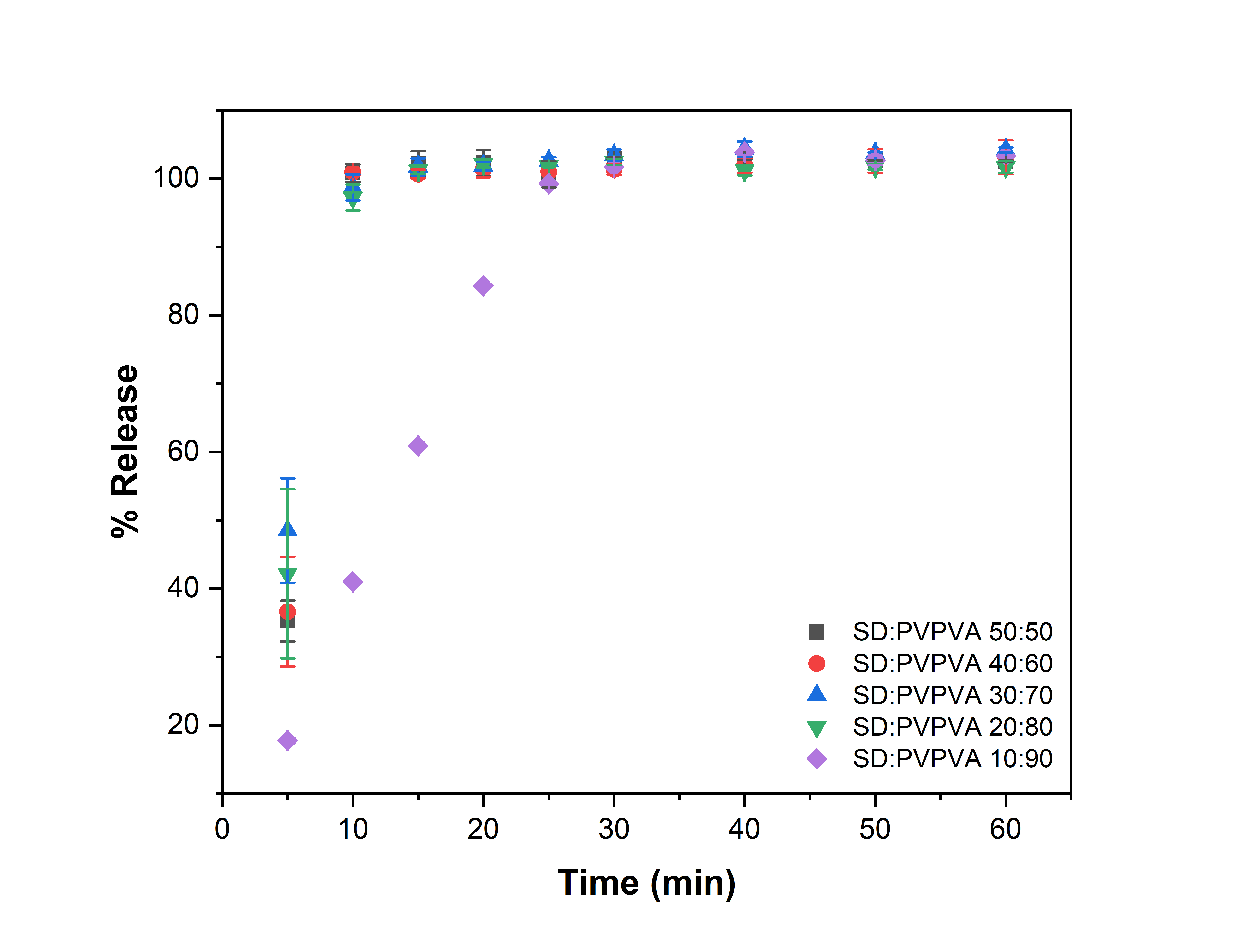


**Figure S2.** **Percent release vs. time profiles for surface normalized dissolution for PVPVA from various w/w ratios of SD:PVPVA dispersions. Error bars represent standard deviation, n = 3.**


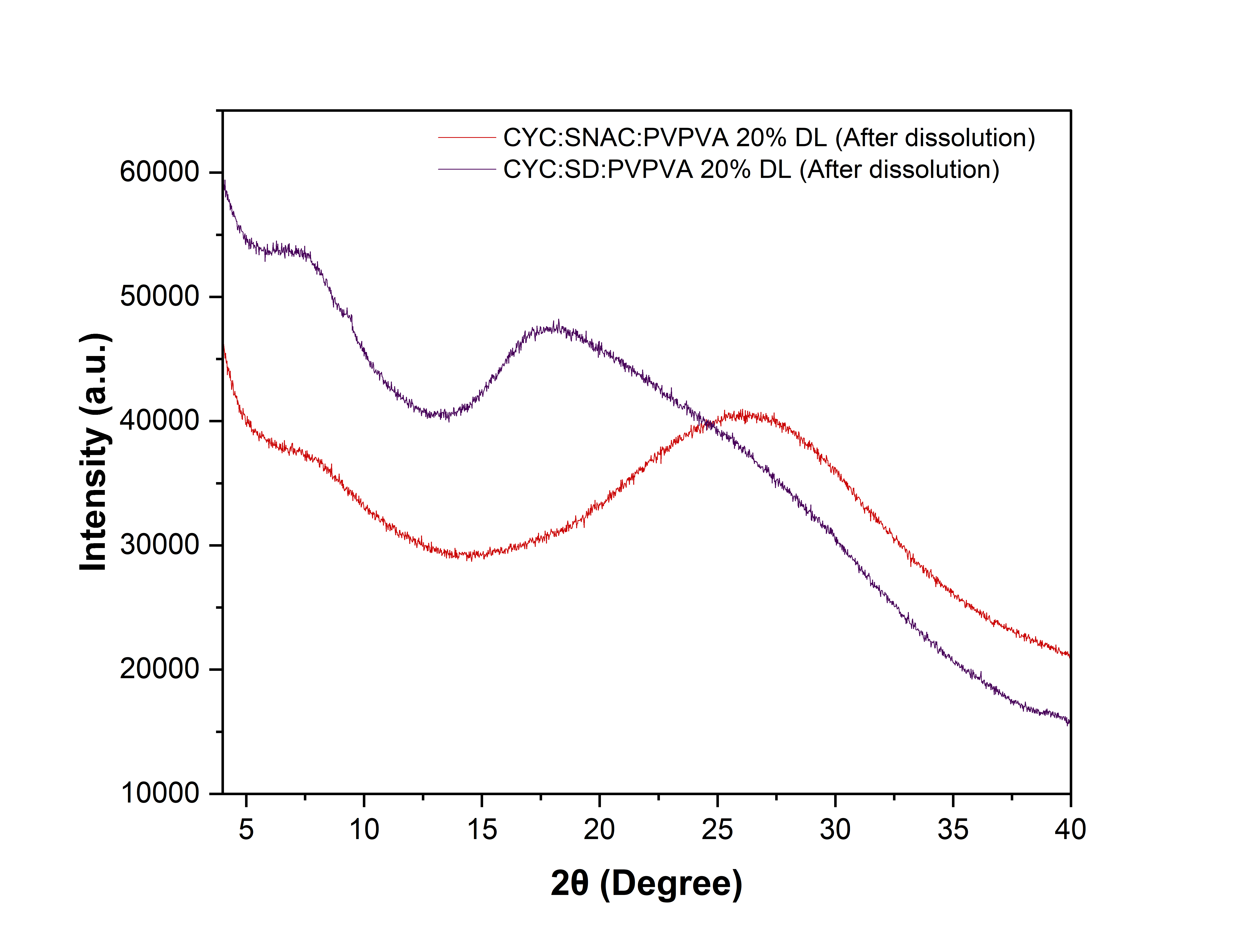


**Figure S3. PXRD diffractogram of CYC: SNAC: PVPVA and CYC:SD: PVPVA 20% DL dispersions after partial dissolution.**
